# Supplementary figures and images for: Group B Streptococcus-Induced Macropinocytosis Contributes to Bacterial Invasion of Brain Endothelial Cells
Source: Pathogens. 2022 Apr 15;11(4):474. doi: 10.3390/pathogens11040474 (PMC9028350; doi:10.3390/pathogens11040474)

**A**

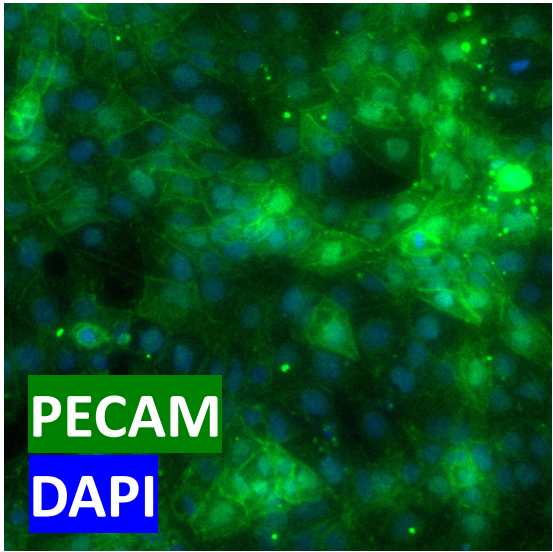

**B**

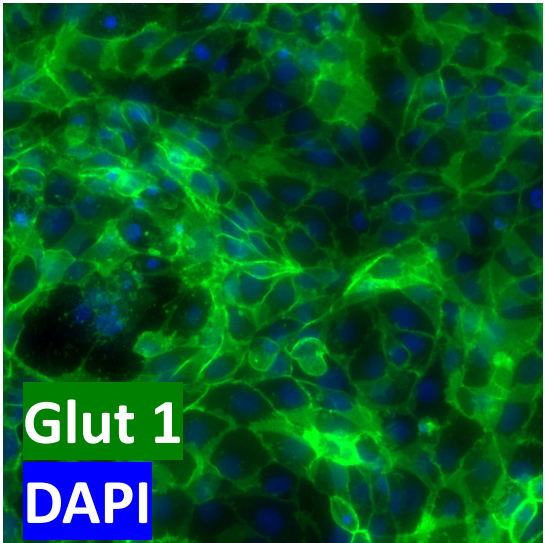

**C**

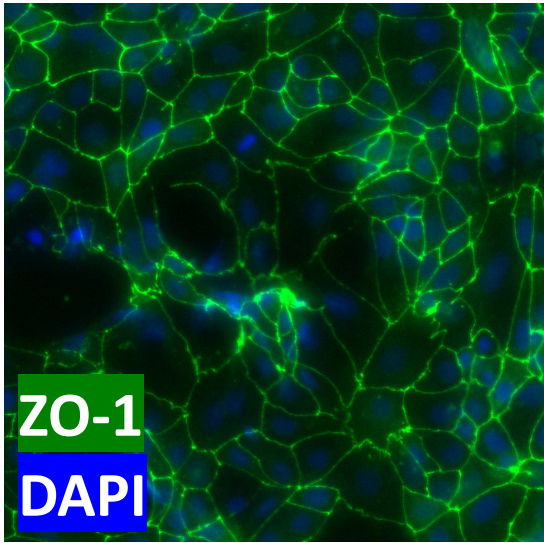

**D**

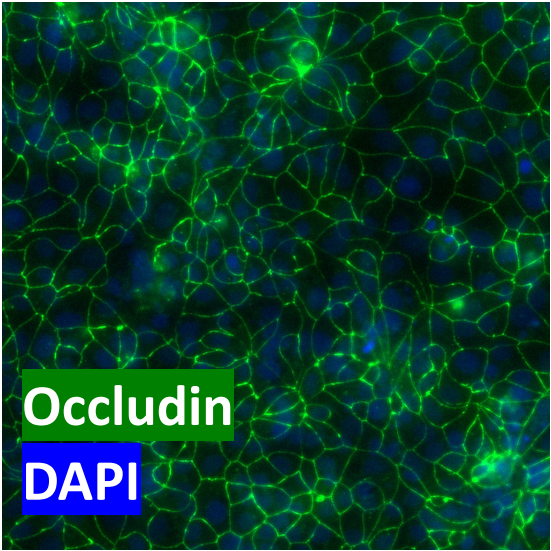

**E**

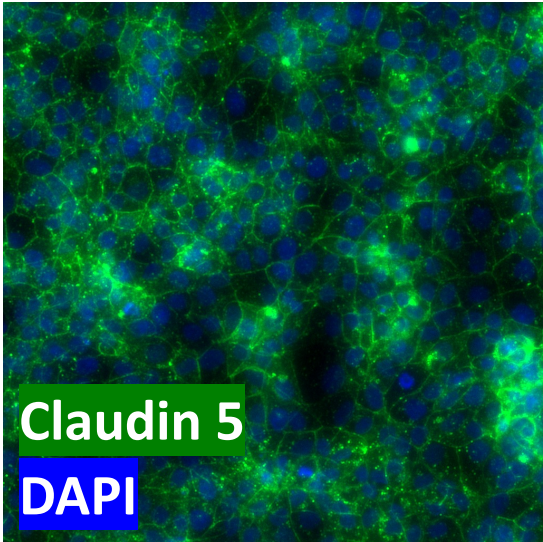

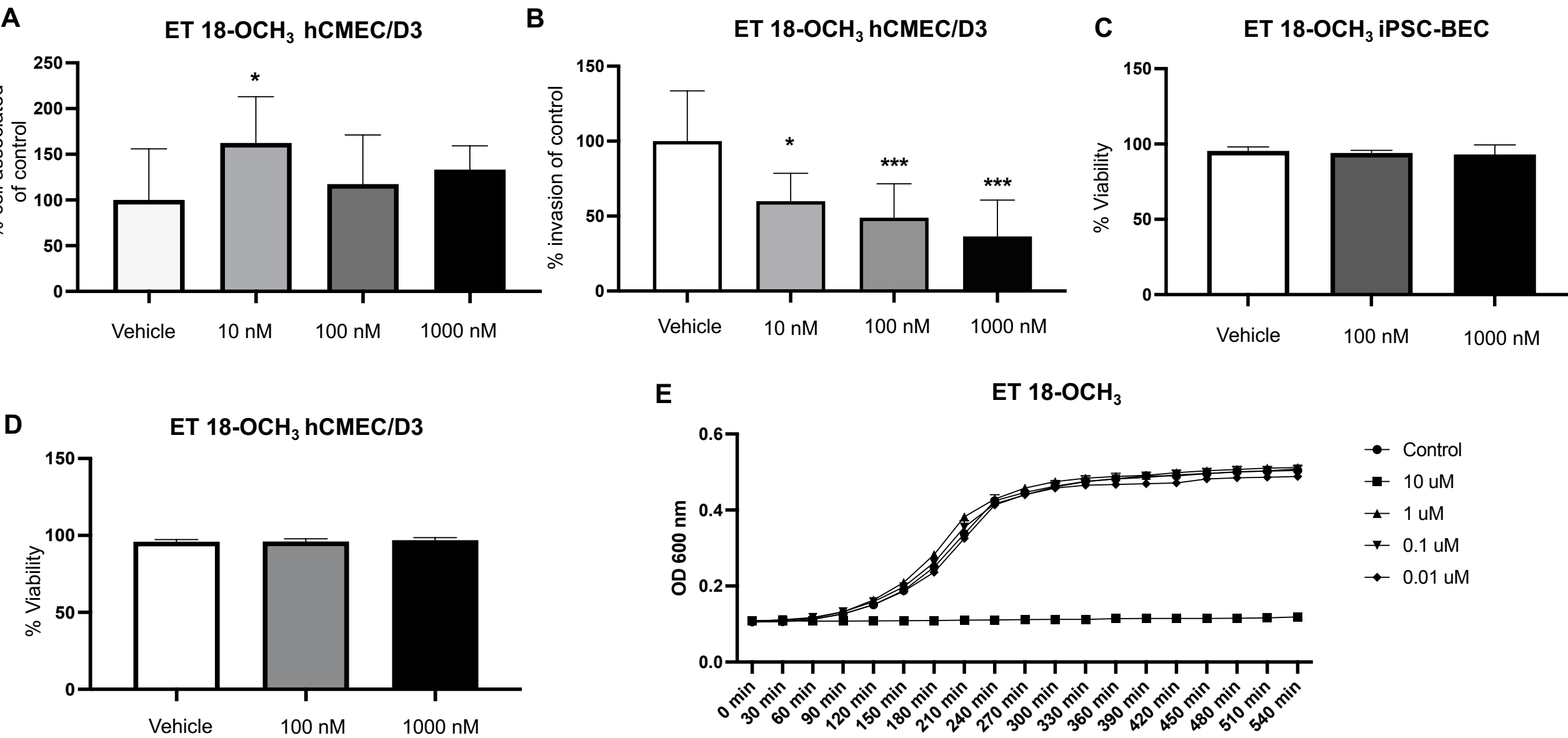

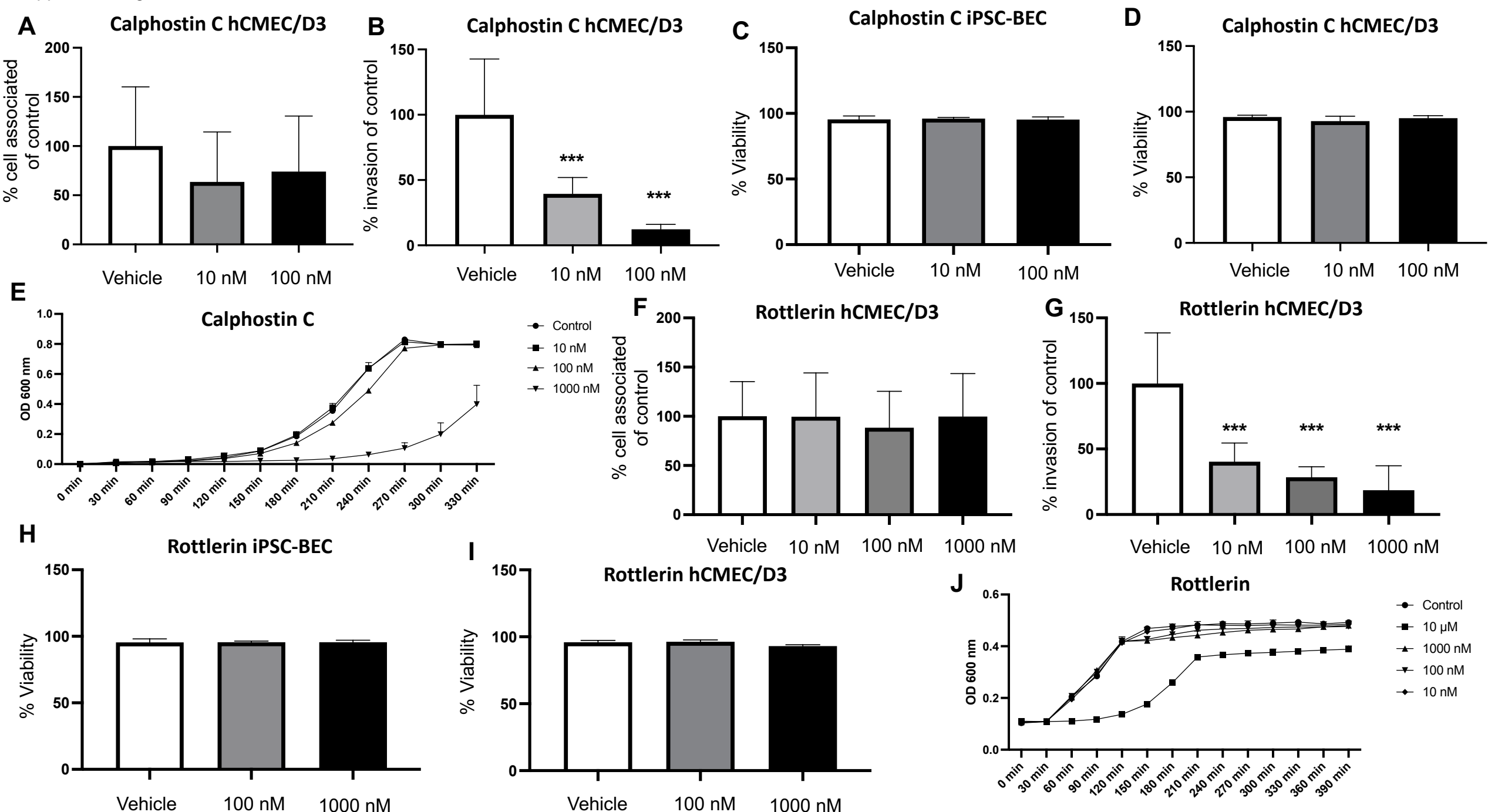

Supplement Figure S4

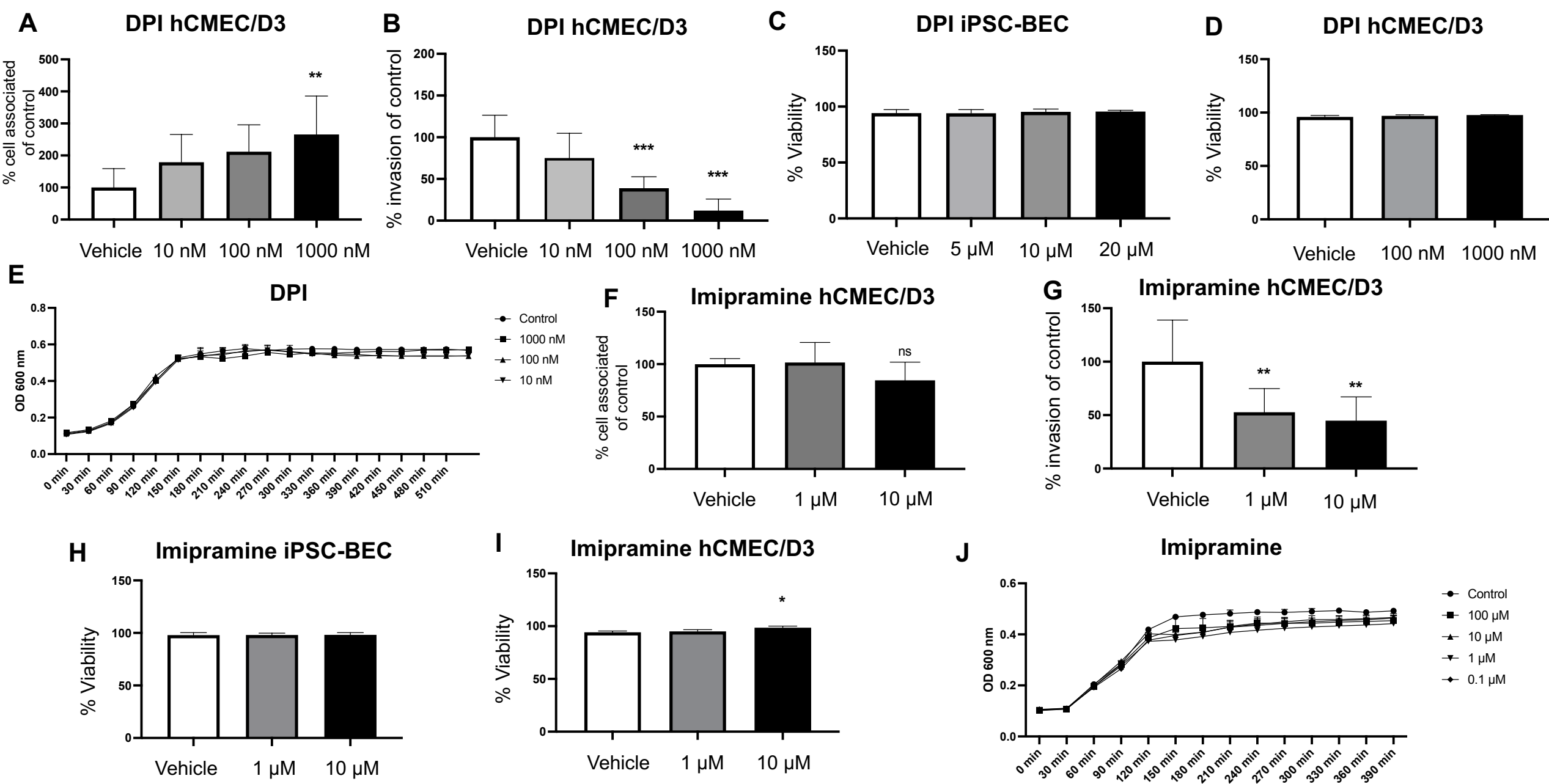

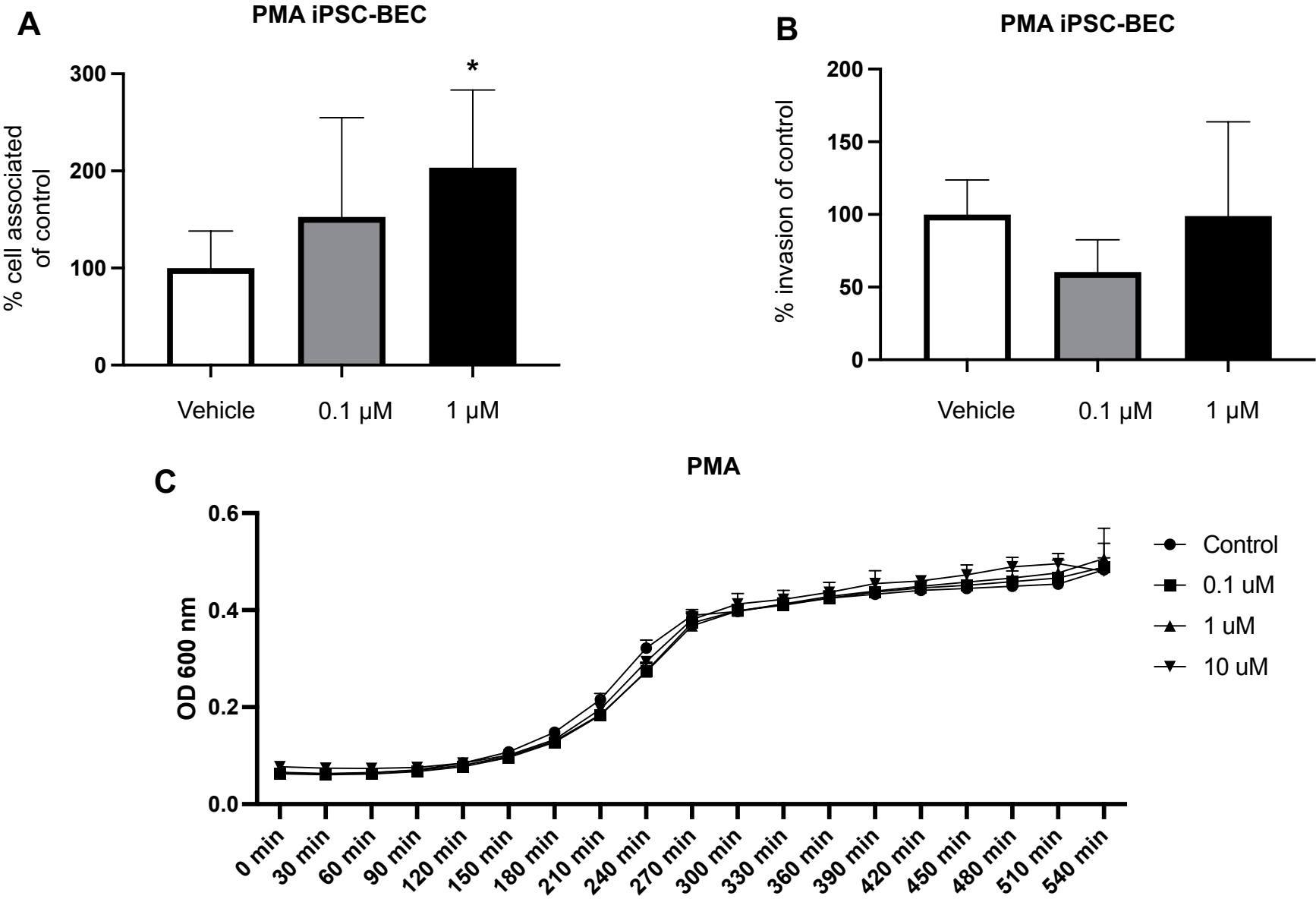

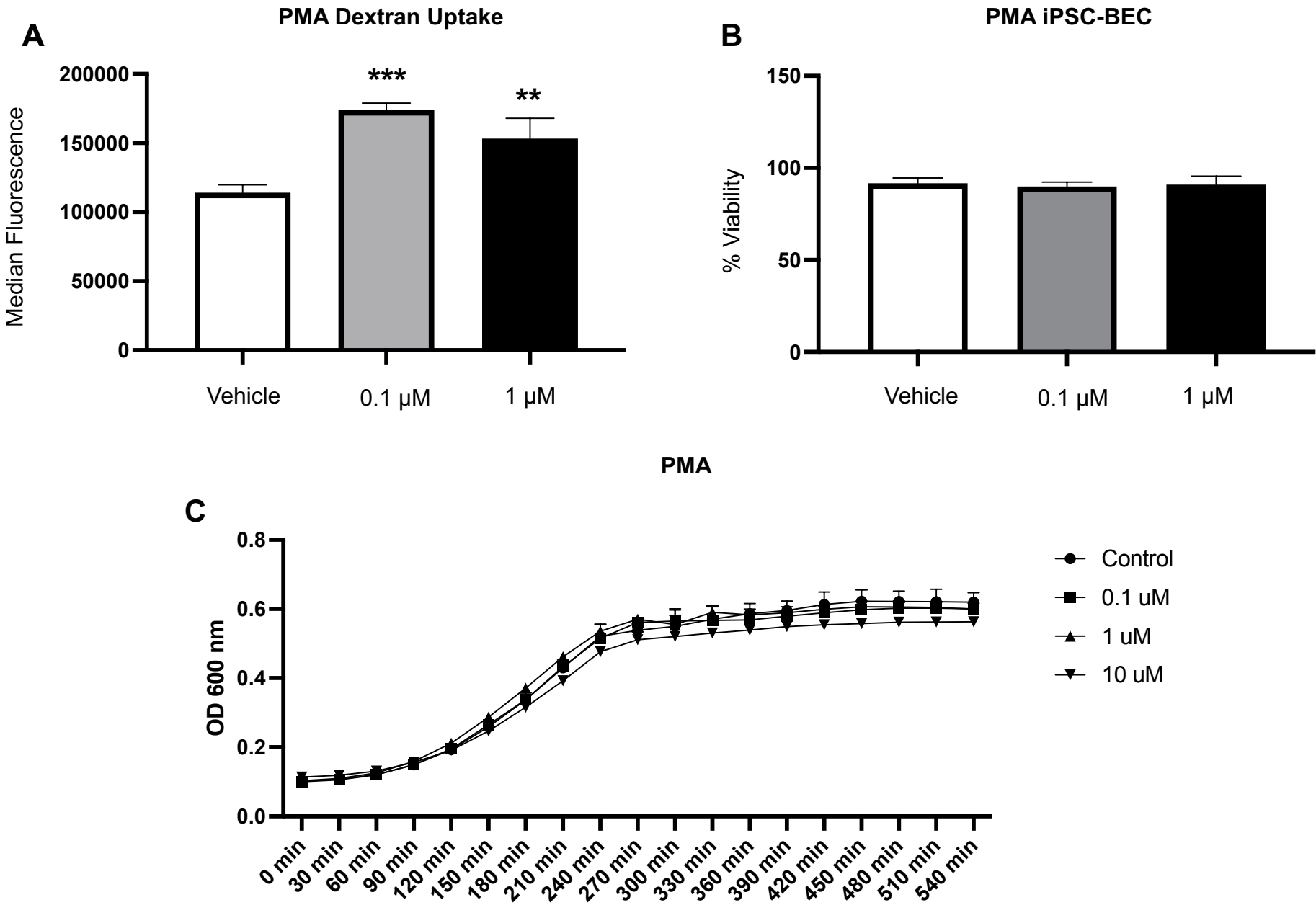

Supplement: Supplementary file 1 [file pathogens-11-00474-s001.zip › pathogens-1644644-supplementary.pdf]
